# Health inequities and cancer survival in Manizales, Colombia: a population-based study

**DOI:** 10.25100/cm.v49i1.3629

**Published:** 2018-03-30

**Authors:** Nelson Enrique Arias-Ortiz, Esther de Vries

**Affiliations:** 1 Departamento de Salud Pública, Universidad de Caldas, Manizales, Colombia.; 2 Departmento de Epidemiología Clinica y Bioestadistica, Pontificia Universidad Javeriana, Bogotá, Colombia

**Keywords:** malignant neoplasms, survival analysis, socioeconomic factors, insurance, health, neoplasias malignas, análisis de sobrevida, factores socioeconómicos, aseguramiento

## Abstract

**Objective:**

To analyze differences in survival of breast, cervical, lung, prostate and stomach cancer by health insurance regime (HIR) and socioeconomic position (SEP) in an intermediate city in a middle-income country.

**Methods:**

All patients with breast, cervix uteri, lung, prostate and stomach cancer diagnosed between 2003 and 2007 and characterized by the Manizales population-based Cancer Registry (MCR) were included and followed up to a maximum of 5 years for identifying deaths. Survival probabilities estimated by HIR were defined according to the type of affiliation at the date of diagnosis, and by socioeconomic stratification of residence (SS) as indicator of SEP, stratifying for other prognostic factors using Kaplan-Meier methods. Cox proportional hazard models were fitted for multivariate analysis.

**Results:**

A total of 1,384 cases and 700 deaths were analyzed**.** Five-year observed survival was 71.0% (95% IC: 66.1-75.3) for breast, 51.4% (95% IC: 44.6-57.9) for cervix, 15.4% (95% IC: 10.7-20.8) for lung, 71.1% (95% IC: 65.3-76.1) for prostate and 23.8% (95% IC: 19.3-28.6) for stomach. Statistically significant differences in survival by HIR were observed for breast, lung, prostate, and stomach - with poorer survival for the subsidized and uninsured patients. Differences by SS were observed for lung and prostate. Differences in survival by HIR were independent of SS, and viceversa.

**Conclusions:**

Important inequities in cancer survival exist related to HIR and SEP. Possible explanations include underlying comorbidities, late stage at diagnosis, or barriers to timely and effective treatment.

## Resumen

**Objetivo:**

Analizar la supervivencia de pacientes con cáncer de mama, cuello uterino, pulmón, próstata y estómago según régimen de aseguramiento en salud (RAS) y posición socioeconómica (PSE) en una ciudad intermedia de un país de medianos ingresos.

**Métodos:**

Se incluyeron todos los pacientes con cáncer de mama, cuello uterino, pulmón, próstata y estómago diagnosticados entre 2003 y 2007 y caracterizados por el Registro Poblacional de Cáncer de Manizales, quienes fueron seguidos hasta un máximo de cinco años para identificar los fallecimientos. Las probabilidades de supervivencia estimada según RAS fueron definidas de acuerdo con el tipo de afiliación al momento del diagnóstico, y según el estrato socioeconómico de la residencia como indicador de PSE, estratificando por otros factores pronósticos y utilizando el método de Kaplan-Meier. Para el análisis multivariado se ajustaron modelos de riesgos proporcionales de Cox.

**Resultados:**

Se analizaron en total 1.384 casos y 700 muertes. La supervivencia observada a cinco años fue 71.0% (IC 95%: 66.1-75.3) para cáncer de mama, 51.4% (44.6-57.9) para cuello uterino, 15.4% (10.7-20.8) para pulmón, 71.1% (65.3-76.1) para próstata, y 23.8% (19.3-28.6) para estómago. Se observaron diferencias estadísticamente significativas en la supervivencia según RAS para mama, pulmón, próstata y estómago, con supervivencia más pobre en los pacientes del régimen subsidiado y no asegurados. Se observaron diferencias por estrato socioeconómico en los cánceres de pulmón y próstata. Las diferencias por RAS fueron independientes del estrato socioeconómico y viceversa.

**Conclusiones:**

Existen importantes inequidades en la supervivencia de pacientes con cáncer relacionadas con el RAS y con la PSE. Las posibles explicaciones incluyen comorbilidades subyacentes, diagnóstico tardío y barreras para el acceso al tratamiento oportuno y efectivo.

## Introduction

Socio-economic differences in cancer survival have been well documented in the last two decades ^1^. Disparities in stage at diagnosis have been considered as the main underlying factor of those differences. However, there is evidence that differential access to timely and adequate treatment by socio-economic groups also determines cancer survival inequities ^1^. Estimations of relative risk of dying comparing the most deprived groups with the best-off groups have been around 1.3 to 1.5-fold; factors underlying these differences include tumour and patient conditions, and access to and quality of care ^1^. In countries with universal health care insurance like Canada, socioeconomic position (SEP) remained associated with cancer survival, the differences are not being due to stage at diagnosis ^2^. Inequalities and inequities have been reported for all cancer sites combined ^1^, but also for specific sites including breast ^3^, cervix ^4^, lung ^5^, prostate ^6^ and stomach ^7^ ^,^ ^8^ cancers. For breast and cervical cancer, large differences were observed between developed and developing countries but also between developed countries and within countries ^9^. For female cancers, place of residence, income, socioeconomic and educational level, ethnicity, and migration status have been associated with inequities in survival ^9^.

In Colombia, health insurance financed through contributions of both workers and employers (contributory regime) is mandatory for dependent employees and partially voluntary for independent workers. A small proportion (<5%) of the population, working in certain public sectors, has exceptional or special health insurance plans (special or exceptional regime). The poor population is covered by a subsidized health system founded through taxes (subsidized regime) ^10^. In 2005, contributory and subsidized regimes covered 36.3% and 43.3% of the population, respectively, while about 20% of population had no health insurance^11^. In theory, special and contributory regimes offer the best access to care, but in practice special/exceptional regimes have shown some problems that could make the conditions of its affiliates worse than those of the contributory regime^12^ ^,^ ^13^. Recent laws (years 2011^14^ and 2015^15^) have advocated for universal health care access without differences by SEP. However, those legislative changes have not been fully implemented, and patients diagnosed prior to 2010 may have experienced different survival rates depending on their health insurance status and their SEP. Large socioeconomic disparities in gastric cancer survival were recently documented in other cities in Colombia, despite improvements in health insurance coverage ^8^ and therefore it is not surprising that large inequities in population-based cancer mortality exist ^17^ ^,^ ^18^. Hardly any population-based information by socioeconomic indicators is available for other cities and other cancers in Colombia.

Manizales is a middle-size Andean city in Colombia with a projected population in 2005 (mid-term year) of 379,794 inhabitants ^18^. Since 2003, the city hosts a population-based cancer registry that meets international standards proposed by IARC^19^ and has reported incidence rates that are slightly higher than national estimations, mainly for breast, lung and stomach cancers ^20^ ^,^ ^21^ - coinciding with a higher smoker rate and position in the Andes. With incidences known, there is a need to evaluate population-based survival for this population, and evaluate the effect of health insurance and SEP on its survival rates. The aim of this paper is to analyze differences in breast, cervical, lung, prostate and stomach cancer survival as a result of health insurance and socioeconomic status in this intermediate, Andean city in Colombia.

## Materials and Methods

### Type of study

Exploratory population-based cohort study. 

### Patients and follow up

All 1,482 patients resident in Manizales with breast, cervix uteri, lung, prostate or stomach cancer diagnosed between 2003 and 2007 and characterized at baseline by Manizales’ Cancer Registry - MCR (information regarding data quality of MCR was published previously^19^ ^,^ ^22^ were included: 380 female-breast cancer cases (5 male cases were excluded), 226 cervix uteri, 230 lung, 296 prostate, and 347 stomach cancer patients. The percentages of microscopically verified cases were 95.8%, 96.9%, 78.6%, 92.5% and 89.4% for breast, cervix, lung, prostate, and stomach, respectively. 

All cases were followed up until 60 months or until December 31th 2013 since diagnosis for identifying the event of study (deaths due to all causes) and time to event through matching personal identity numbers and names of incident cases with the local vital statistics provided by the local health authority; also, we performed manually searching in electoral rolls and health insurance databases. Active follow-up was performed by consultation of medical records where available. When only data for year was available, month and day were assigned to June 30^th^. Patients without event were censored at five years of follow-up. Subjects were considered alive if they were eligible to vote or if they were reported as “active” in health insurance databases on December 31th 2013. Survival time was calculated as the difference between incidence date and date of death, date of last contact with health system, date of loss to follow up, or date they were censored. For incidence data, MCR uses rules from European Network of Cancer Registries ^23^.

Sixteen subjects with a clinical cancer diagnosis were lost to follow up at day of diagnosis and were therefore treated the same as DCO cases. There were 77 cases identified only by their death certificate (DCO), representing 5.2% of all cases (breast: n= 5 (1.3%); cervix uteri: n= 5 (2.2%); lung: n= 29 (12.6%); prostate: n= 19 (6.4%) and stomach: n= 19 (5.5%). According to international registry standards, a case is flagged as DCO when death certificate is the only source of data for the case, i.e, there is no other data from pathological reports, medical images, or hospitals. Since DCO cases by definition do not have information about time to event, they were excluded from survival analyses.

### Clinical and demographic characteristics

Information on histological subtype coded according to the International Classification of Diseases for Oncolgy, 3^rd^ revision -ICDO-3- was available for 95.2% of cases; clinical stage at diagnosis according to TNM system was available in usable proportions of patients only for breast (62%) and cervix (42%) cancer. For other sites, clinical stage was available in less than 30% of cases and could therefore not be used. There were no patients with missing data for age at diagnosis. More than 85% of patients had complete date of birth, and their ages in clinical records were consistent with calculated ages. For patients without date of birth, age in clinical records was assumed as correct.

Differences in diagnostic methods were observed only for prostate and stomach cancers, which “only clinical” and “clinical procedures” methods were observed only for patients in contributory and subsidized regimes and uninsured, while 100% of the cancers diagnosed in patients affiliated to the special regime were histologically confirmed.

### Socioeconomic indicators

Variables for socioeconomic position and health insurance were defined following categories previously used for a Colombian population by de Vries *et al* ^8^. Socioeconomic stratum (SS) of the place of residence at diagnosis was used as indicator of SEP. In Colombia, SS is defined according to external and internal physical characteristics of dwellings and wards, ranging from the purely functional and indispensable to the aesthetic, ornamental and sumptuous characteristics. SS is reported in categories from 1 to 6, where 1 and 2 corresponds to “low” social stratum, 3 and 4 to “middle”, and 5 and 6 to “high”.

Health Insurance Regime (HIR) at the date of diagnosis was used grouping the special and exceptional regimes into one unique category, contributory regime, subsidized regime, and a group of uninsured people. 

### Statistical analysis 

Observed survival proportions at different times were obtained using Kaplan-Meier analyses, stratifying analyses by HIR and SS, age, sex, histological subtype and, for breast and cervical cancer only, clinical stage at diagnosis. Cox multivariate proportional hazard assumption was checked by visual evaluation of log-log plots; the assumption was not violated. Three Cox multivariate regression models for each cancer were fitted for both HIR and SS: i) a univariate (null) model; ii) a multivariate model A with age, sex (lung and stomach), histological subtype, and clinical stage (breast and cervix) as covariates ; and iii) a model B containing all variables of model A plus an additional term for SS in the HIR model and vice versa ^8^. All calculations were performed using STATA™ SE 12.0.

### Ethical considerations

This research was approved by the Research Ethics Committees of the Universidad del Valle, Universidad de Caldas, and the National Cancer Institute of Colombia.

## Results

Patient and tumour characteristics for all 1,405 incident cases are shown in Supplementary Table 1S.

For the five cancer sites studied, 1,384 cases were finally analyzed. Lost of follow-up was 1.7% for five sites studied (0.8% for breast, 2.3% for cervix, 1.9% for lung, 1.8% for prostate, and 2.1% for stomach). In Manizales, HIR coverage among cancer patients was 88.8%, except for gastric cancer, in which 18.3% had no affiliation to HIR. Breast and prostate patients without HIR tended to be older at diagnosis than affiliated, but differences were not statistically significant. 

At five-years follow-up, 700 deaths (all causes) were observed. Mean follow-up time for overall sites was 38.4 months (95% CI: 37.2; 39.7), varying from 18.5 months for lung to 50.8 months for breast cancer. Five-year observed survival (OS) was 49.1% (95% CI: 46.4-51.7) for the five sites combined. By cancer site, 5-year OS were 71.0% (95% IC: 66.1-75.3), 51.4% (95% CI: 44.6-57.9), 15.4% (95% CI: 10.7-20.8), 71.1% (95% CI: 65.3-76.1) and 23.8% (95% CI: 19.3-28.6) for breast, cervix uteri, lung, prostate, and stomach, respectively (Table 1). 

**Table 1:** Survival estimations by cancer site and prognostic factors. Manizales, 2003-2013

		Cases (n)	Deaths (n)	Proportion surviving after (%)			WBG test*
				12 m	36 m	60 m	
Breast	All cases	375	108	93.8	81.0	71.0	
	Age at diagnosis						
	0 a 49	116	29	96.5	85.2	74.8	X^2^=1.63
	50+	258	79	92.6	79.1	69.3	p=0.20
	Histology						
	Ductal Ca.	307	82	95.1	83.1	73.3	**X** ^**2**^ **=6.19**
	Other and NOS	67	26	87.9	71.2	60.4	**p=0.013**
	Clinical stage						
	Stage I	25	0	100.0	100.0	100.0	**X** ^**2**^ **=80.76**
	Stage II	97	5	100.0	96.9	94.9	**p=0.000**
	Stage III	82	19	100.0	89.0	76.8	
	Stage IV	29	11	93.1	69.0	55.2	
	Unknown	141	71	85.0	64.3	49.2	
Cervix	All cases	220	105	80.7	62.1	51.4	
	Age at diagnosis						
	0 a 49	101	42	83.0	68.0	58.0	X^2^=2.40
	50+	119	63	78.7	57.1	45.8	p=0.121
	Histology						
	Squamos cell Ca.	167	80	80.1	60.7	51.6	X^2^= 1.99
	AdenoCa.	38	16	83.9	70.3	56.8	p=0.369
	Other and NOS	15	9	79.4	57.8	36.1	
	Clinical stage						
	Stage I	16	2	100.0	93.8	87.5	**X** ^**2**^ **= 13.75**
	Stage II	36	16	91.7	69.4	55.6	**p=0.008**
	Stage III	30	19	80.0	53.3	36.7	
	Stage IV	13	10	69.2	38.5	23.1	
	Unknown	125	58	76.3	60.5	52.2	
Lung	All cases	198	165	43.6	21.0	15.4	
	Sex						
	Women	81	67	50.6	28.4	17.3	X2= 3.25
	Men	117	98	38.7	15.8	14.1	p=0.071
	Age at diagnosis						
	0 a 59	59	46	40.7	27.1	22.0	X2=0.94
	60+	139	119	44.9	18.4	12.5	p=0.331
	Histology						
	Squamous cell Ca.	71	54	49.3	22.5	16.9	X2=5.96
	AdenoCa.	53	44	37.3	17.7	13.8	p=0.113
	Small cell Ca.	16	13	56.3	31.2	18.7	
	Other and NOS	58	49	38.6	19.3	14.0	
Prostate	All cases	270	78	92.2	78.9	71.1	
	Age at diagnosis						
	0 a 59	43	8	95.4	83.7	81.4	*X*2=2.05
	60+	227	70	91.6	77.8	69.2	p=0.152
	Histology						
	Adeno Ca.	257	70	92.3	79.4	72.2	*X*2=2.30
	Other and NOS	18	8	83.3	72.2	55.6	p=0.129
Stomach	All cases	322	244	49.4	33.1	23.8	
	Sex						
	Women	114	83	57.6	39.0	26.6	X2=1.31
	Men	208	161	44.9	30.0	22.2	p=0.252
	Age at diagnosis						
	0 a 59	112	86	48.2	32.1	23.2	X2=0.03
	60+	210	158	50.0	33.7	24.1	p=0.857
	Histology						
	Adeno Ca, intestinal	146	111	54.5	33.8	23.5	X2=2.01
	Difuse Ca.	73	59	41.7	26.4	18.1	p=0.569
	Adeno Ca., others	48	35	47.9	37.5	27.1	
	Other and NOS	55	39	47.3	36.4	29.1	

NOS: Non other specification *WBG: Wilcoxon -Breslow-Gehan test.

**Figure 1:**
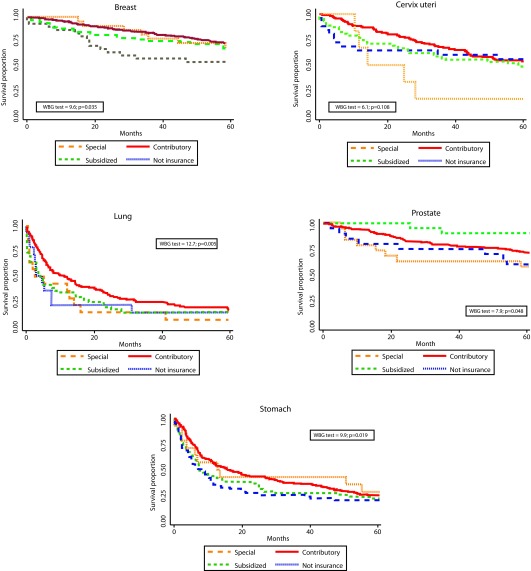
Observed survival by health insurance regime and cancer site. Manizales, 2003-2013. WBG: Wilcoxon-Breslow-Gehan test.

**Figure 2:**
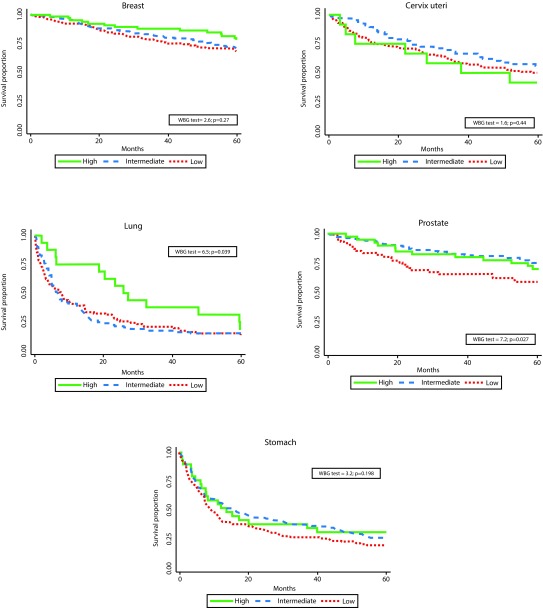
Observed survival by social strata and by cancer site. Manizales, 2003-2013. WBG: Wilcoxon-Breslow-Gehan test.

Statistically significant differences in survival by HIR were observed for breast, lung, prostate, and stomach - with poorer survival for the subsidized and uninsured patients. Differences by SS were observed for lung and prostate. (Figs. 1  and 2). One and five-years OS proportions were significantly lower in uninsured or subsidized patients versus patients with special or contributory HIR, with exception of cervix uteri cancer. Regarding SS, differences statistically significant were only observed in lung and prostate cancers, with poorer survival proportions in patients for low/middle versus high SS and low versus middle/high SS, respectively. However, survival proportions for cervix uteri and lung cancer of patients affiliated to the special/ exceptional HIR were lower than in the other categories, even lower than in uninsured population.

**Figure 3:**
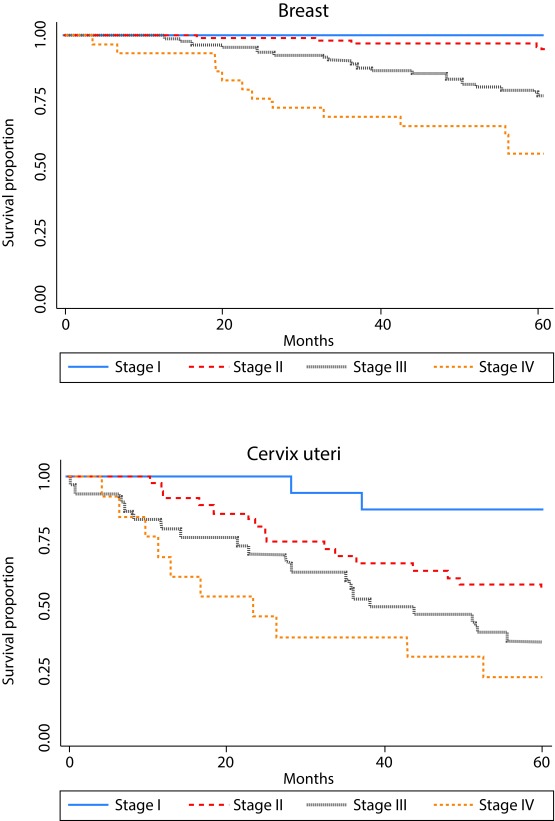
Survival proportion by clinical stage at diagnosis for breast cancer (based on 233 cases with known clinical stage) and cervix uteri cancer (based on 95 cases). Manizales, 2003-2013.

As expected, overall survival was higher in younger patients for all sites studied, but those differences were not statistically significant. According to literature, clinical stage at diagnosis showed a strong association with survival (Table 1 and Fig. 3). Survival was better for women diagnosed with ductal breast carcinoma vs. other histological subtypes. Non-significant differences in survival were observed by histological subtypes of cervix, lung, prostate and stomach cancers. For lung and stomach cancers no survival differences by sex were observed.

Table 2 shows results from Cox models by HIR and SS and by cancer site. For prostate cancer, HIR hazard ratios (HR) remained significant after adjusting for age and histological subtype, with lower hazard of dying for special HIR group in comparison with the subsidized regime (HR: 0.17 (95% IC: 0.04-0.80)). These results remained significant in multivariate analyses. For stomach cancer, patients in contributory regime had better survival in all, univariate and multivariate, models, with about 30% lower hazard of dying in comparison with patients in subsidized HIR. With respect to socioeconomic position, prostate cancer patients from middle SS showed about 47% lower hazard of dying than patients from low SS (HR 0.53, 95% CI: 0.31-0.88), independently of health insurance regime. Other sites did not reach statistical significance, possibly due to the low number of cases in each group. 

**Table 2:** Proportional risks (Cox) survival models by cancer site.

		Univariate analysis		Multivariate analysis			
		HR	95% CI	Model A		Model B	
				HR	95% CI	HR	95% CI
Breast cancer	Health insurance *						
	Contributory	0.77	0.47-12.7	0.71	0.43-1.17	0.77	0.44-1.36
	Special	0.89	0.38-2.09	0.47	0.20-1.13	0.52	0.20-1.34
	Not insured	1.66	0.84-2.26	1.27	0.64-2.55	1.41	0.65-3.07
	Cases (events)	370 (108)		370 (108)		345 (99)	
	Social Strata ^a^						
	Middle	0.88	0.57-1.35	0.75	0.48-1.17	0.93	0.56-1.55
	High	0.61	0.33-1.14	0.65	0.35-1.21	0.84	0.42-1.65
	Cases (events)	347 (99)		347 (99)		345 (99)	
Cervical cancer	Health insurance *						
	Contributory	0.78	0.51-1.22	1.76	0.85-3.64	2.12	0.99-4.55
	Special	2.20	0.85-5.68	**5.02**	**1.69-14.9**	**7.60**	**1.94-29.7**
	Not insured	0.92	0.46-1.81	0.94	0.25-3.58	0.90	0.23-3.51
	Cases (events)	217 (104)		92 (46)		87 (43)	
	Social Strata ^a^						
	Middle	0.78	0.50-1.22	0.91	0.47-1.76	0.67	0.32-1.41
	High	1.20	0.55-2.62	0.84	0.19-3-65	0.38	0.08-1.87
	Cases (events)	197 (95)		88 (44)		87 (43)	
Lung cancer	Health insurance *						
	Contributory	0.69	0.47-1.02	0.75	0.51-1.12	0.89	0.58-1.36
	Special	1.15	0.61-2.19	1.22	0.64-2.32	1.28	0.66-2.49
	Not insured	1.04	0.54-2.01	1.32	0.66-2.62	1.77	0.83-3.77
	Cases (events)	197 (165)		197 (165)		186 (157)	
	Social Strata ^a^						
	Middle	1.00	0.72-1.39	0.99	0.71-1.38	1.03	0.74-1.46
	High	0.58	0.32-1.05	0.60	0.33-1.09	0.67	0.36-1.24
	Cases (events)	186 (157)		186 (157)		186 (157)	
Prostate cancer	Health insurance *						
	Contributory	0.58	0.28-1.21	0.56	0.27-1.18	0.56	0.26-1.21
	Special	**0.17**	**0.04-0.80**	**0.20**	**0.04-0.95**	**0.12**	**0.02-0.99**
	Not insured	0.89	0.33-2.37	0.74	0.27-2.02	0.81	0.27-2.41
	Cases (events)	266 (77)		266 (77)		233 (70)	
	Social Strata ^a^						
	Middle	**0.52**	**0.31-0.87**	**0.53**	**0.31-0.88**	**0.54**	**0.32-0-92**
	High	0.63	0.32-1.26	0.71	0.36-1.44	0.78	0.38-1.60
	Cases (events)	237 (71)		237 (71)		233 (70)	
Stomach cancer	Health insurance *						
	Contributory	**0.72**	**0.52-0.98**	**0.71**	**0.52-0.98**	**0.70**	**0.51-0.98**
	Special	0.70	0.36-1.38	0.72	0.36-1.41	0.75	0.38-1.50
	Not insured	0.93	0.62-1.39	0.93	0.62-1.39	1.01	0.65-1.56
	Cases (events)	320 (243)		320 (243)		289 (221)	
	Social Strata ^a^						
	Middle	0.80	0.61-1.06	0.80	0.60-1.05	0.82	0.62-1.09
	High	0.75	0.46-1.20	0.76	0.47-1.23	0.89	0.55-1.45
Cases (events)	291 (222)		291 (222)		289 (221)	

* Subsidized regime as reference a Low strata as reference. Models were adjusted by age, sex (lung and stomach), histological subtype, and stage at diagnosis (breast and cervix).

Unsurprisingly, advanced clinical stages for breast and cervix had increased HRs. In line with the Kaplan-Meier results, cervical cancer patients affiliated to the special regime had a higher hazard than women affiliated to the subsidized regime after adjusting by age, histological subtype and clinical stage. 

Model B showed that inclusion of both terms HIR and SS in the same model modified HR estimates in all cancers combined and by cancer site, indicating independent effects of HIR and SS on survival.

## Discussion

This population-based study on population-based survival for five cancer sites in Manizales, Colombia, demonstrated significant differences in observed survival. Differences by HIR varied from 8 percent-points in stomach to 32 percent-points in prostate cancer. Hazard Ratios estimated for HIR were in line with risks reported in other studies ^1^. Absolute differences by SS were less noticeable, with differences between low versus high categories of about 16 percent-points for prostate, 10 for breast, 14 for cervix, 5 for lung, and 11 percent-points for stomach cancers. The magnitude of these disparities is similar with those found in U.S for the last quarter of the past century ^24^.

Colombian health system was radically reformed at the end of the last century, resulting in a substantial increase in coverage of health insurance which reached almost 100% around 2010. On paper, this meant a substantial improvement in access to health services. However, timely access to health care in cancer diagnosis and treatment is still problematic, particularly because of the high out-of-pocket cost and long waiting times to obtain permission to use these services. In Colombia, access to health care is differential according to the health insurance regime, and inequities persist between types of affiliation. Local researchers have pointed out that universality in National Health System has not been achieved and there has been a stagnation in matters regarding access to services and equality^25^. Additionally, enormous regional disparities have been described in Colombia, and the country has one of the worst distributions of per capita income in the world ^26^. 

In general, 5-year OS by site was below that observed for most countries in the CONCORD ^27^ and EUROCARE^28^ studies. Disparities by HIR and SS for breast, prostate and stomach cancers were similar to reported by literature ^29^ ^-^ ^33^.

The survival proportion for women with a cancer of the cervix uteri was 3-fold lower among women affiliated to the special regime compared to the other HIR groups. This surprising results - special regimes have, in theory, the most generous health care plan - are in line with the observation of the worst stage at diagnosis in this group, which suggest that screening and early detection programs are not properly working in special regime entities. Regarding socioeconomic stratification, survival rates or cervical cancer were 7 and 14 percent-points higher in low and middle social strata, respectively, in comparison with the richest group. Incidence rates were lower in the richest group, and the relative low frequency of disease among the wealthiest part of the population may result in a lower awareness or lower participation rates in screening and early treatment programs for cervical cancer. However, differences in clinical stage at diagnosis did not reach statistical significance - perhaps because number of cases in the high strata was very low (see supplementary table). In this regard, Brookfield *et al*. ^34^, found that, in women living in the state of Florida (USA), the independent predictors of poorer outcomes were insurance status, tumor stage, tumor grade, and treatment. Neither race, nor ethnicity, nor SES was an independent predictor of poorer outcome. Similarly, Niu *et al*. ^35^, found no significant differences in cervical cancer survival by insurance status in New Jersey.

For lung cancer, 2.3 and 2-fold better survival rates were observed in patients from contributory, subsidized and non-insured categories in comparison with special regimes. These results are contradictory with those reported in US ^36^ where uninsured and Medicaid patients had poorer survival than patients with private insurance. This pattern may be reflecting barriers to early diagnosis and treatment in this subgroup, which in Manizales is mostly composed by teachers, and army and police members. However, this should be confirmed by studies with a larger number of patients. Survival proportions were around five percent-points lower in the lowest socioeconomic stratum in comparison with the most affluent group, which is consistent with figures reported by Ou *et al* ^37^.

Disparities in cancer survival related to the health system can be attributed to barriers and delays in obtaining diagnostic care, associated with more advanced stages at diagnosis. In Colombia, practically all medical procedures require authorization from the insurer, which in many cases lead to substantial diagnostic and treatment delays and - consequently - to more advanced stages in diagnosis and poorer outcomes ^38^ ^,^ ^39^. Therefore, many people turn to the out-of-pocket payment of some services to avoid delays, but people with low financial resources have no other avenues for access to timely diagnosis and treatment and are subject to the administrative procedures of their insurers.

### Strengths and weaknesses

The population-based nature of this work minimizes selection biases in the estimates and serves as a tool for policy-makers to evaluate access and quality of care. Although numbers of patients are relatively small due to the small population size of Manizales, estimates are sufficiently robust to discern general patterns. A major limitation of this study is the lack of relative or net survival estimations by HIR and SS due to lack of available data on population numbers and life-tables by those variables. The registry had no access to the cause of death, making it impossible to calculate cause-specific survival. For cancers with a very poor prognosis, this is not so problematic as most patients will die due to their cancer. However, for cancers with a relatively good prognosis (e.g. breast and prostate), a substantial proportion of deaths may have been due to other causes of death. Considerable proportions of cases had unknown data about SS, specially for cervix, prostate and stomach cancers (12.2%, 13.1% and 11.0%, respectively). In addition, the percentage of cases with missing data for clinical staging at diagnosis was high for all cancers, except breast and cervical cancer: more than 70% of lung, prostate and stomach cancer cases had no stage information, impeding including this variable in the multivariate analyses. The number of cases was very low in some cancer sites and affected multivariate analysis. Around 5% of patients were excluded from analyses because they had no follow-up time (DCO cases and lost-to-follow-up at date of diagnosis), which theoretically could influence findings, as more DCO cases are expected among the lower socioeconomic groups. However, a recent paper showed that, even though DCO diagnosis is associated with low SS, exclusion or inclusion of DCO cases had no significant impact of hazard ratios for survival by socioeconomic variables ^40^. 

## Conclusions

Important inequities in cancer survival exist in Manizales related to health insurance and socioeconomic position. Differences may be attributed to inequities in comorbidities, stage at diagnosis, or barriers to timely access to effective treatment suggested by differences observed between health insurance regimes. 

## Supplementary

**Table S1:** Sociodemographic and clinical characterization of cancer cases, by site.

	n (%)	Health insurance regime (%)						Socioeconomic level (%)				
		Special/ Exceptional	Contributory	Subsidized	Not insurance	Unknown	p-value (chi^**2**^ )	Low	Middle	High	Unknown	p-value (chi^**2**^ )
**All sites studied**	1,405	79 (5.6)	895 (63.7)	259 (18.4)	158 (11.2)	14 (1)		523 (37.2)	577 (41.07)	167 (11.9)	137 (9.75)	
**Breast**	380	23 (6.05)	253 (66.6)	67 (17.6)	33 (8.7)	4 (1.05)		118 (31.1)	166 (43.7)	67 (17.6)	29 (7.6)	
Sex												
Women	375 (98.7)	23 (6.1)	249 (66.4)	67 (17.9)	32 (8.5)	4 (1.06)	0.560	117 (31.2)	165 (44.0)	65 (17.3)	28 (7.5)	0.281
Men	5 (1.3)	0	4 (80.0)	0	1 (20)	0		1 (20.0)	1 (20.0)	2 (40.0)	1 (20.0)	
Age, median (95% CI)	56.5 (55.0-58.0)	50.0 (46.8-57.7)	56.0 (54.0-57.0)	59.1 (53.3-61.9)	60.2 (56.4-67.3)	59.0 (51.2-84.0)		58.5 (54.0-62.1)	54.0 (51.0-57.0)	58.0 (56.0-65.0)	58.0 (51.0-61.6)	
0 a 49	117 (30.8)	9 (7.7)	82 (70.1)	21 (17.9)	5 (4.3)	0	0.186	37 (31.6)	58 (49.69	14 (12.0)	8 (6.8)	0.111
50+	263 (69.2)	14 (5.3)	171 (65.0)	46 (17.5)	28 (10.6)	4 (1.5)		81 (30.8)	108 (41.1)	53 (20.2)	21 (8.0)	
Histology												
Ductal carcinoma	313 (82.4)	21 (6.7)	207 (66.1)	56 (17.9)	26 (8.3)	3 (1.0)	0.641	95 (30.4)	139 (44.4)	55 (17.6)	24 (7.7)	0.780
Others and NOS	67 (17.6)	2 (3.0)	46 (68.7)	11 (16.4)	7 (10.4)	1 (1.5)		23 (34.3)	27 (40.3)	12 (17.9)	5 (7.5)	
Diagnosis method												
Only clinical	5 (1.3)	0	4 (80.0)	1 (20.0)	0	0	0.588	1 (20.0)	2 (40.0)	2 (40.0)	0	0.141
Clinical procedures	7 (1.8)	0	3 (42.9)	3 (42.9)	1 (14.2)	0		5 (71.4)	1 (14.3)	1 (14.3)	0	
Cytological	12 (3.2)	0	7 (58.3)	4 (33.3)	1 (8.4)	0		6 (50.0)	6 (50.0)	0	0	
Histological	356 (93.7)	23 (6.5)	239 (67.1)	59 (16.6)	31 (.7)	4 (1.1)		106 (29.8)	157 (44.1)	64 (18.0)	29 (8.1)	
Clinical stage												
Stage I	25 (6.6)	1 (4.0)	20 (80.0)	4 (16.0)	0	0	0.931	7 (28.0)	13 (52.0)	5 (20.0)	0	0.843
Stage II	98 (25.8)	4 (4.1)	69 (70.4)	18 (18.4)	6 (6.1)	1 (1.0)		30 (30.6)	43 (43.9)	18 (18.4)	7 (7.1)	
Stage III	84 (22.1)	2 (2.4)	59 (70.2)	17 (20.2)	6 (7.1)	0		31 (36.9)	32 (38.1)	18 (21.4)	3 (3.6)	
Stage IV	29 (7.6)	1 (3.4)	20 (69.0)	4 (13.8)	3 (10.3)	1 (3.4)		8 (27.6)	12 (41.4)	8 (27.6)	1 (3.4)	
Unknown	144 (37.9)	15 (10.4)	85 (59.0)	24 (16.7)	18 (12.5)	2 (1.4)		42 (29.2)	66 (45.8)	18 (12.5)	18 (12.5)	
**Cervix uteri**	221	6 (2.7)	122 (55.2)	63 (28.5)	27(12.2)	3 (1.4)		118 (53.4)	63 (28.5)	13 (5.9)	27 (12.2)	
Age, median (95% CI)	52.0 (48.0-55.0)	59.5 (36.7-64.9)	53.0 (48.0-58.0)	51.0 (47.0-59.0)	46.0 (41.7-55.1)	58.0 (37.0-73.0)		50.0 (47.0-54.1)	58.0 (52.0-60.0)	47.0 (33.0-68.0)	47.0 (41.7-59.9)	
0 a 50	101 (45.7)	2 (2.0)	54 (53.5)	28 (27.7)	16 (15.8)	1 (1.0)	0.474	58 (57.4)	22 (21.8)	7 (6.9)	14 (13.9)	0.090
50+	120 (54.3)	4 (3.3)	68 (56.7)	35 (29.2)	11 (9.2)	2 (1.7)		60 (50.0)	44 (36.7)	6 (5.0)	10 (8.3)	
Histology												
Squamous cell Ca.	168 (76.0)	6 (3.6)	90 (53.6)	49 (29.2)	21 (12.5)	2 (1.2)	0.310	99 (58.9)	47 (28.0)	5 (3.0)	17 (10.1)	**0.002**
Adenocarcinoma	38 (17.2)	0	25 (65.8)	10 (26.3)	2 (5.3)	1 (2.6)		14 (36.8)	14 (36.8)	7 (18.4)	3 (7.9)	
Others and NOS	15 (6.8)	0	7 (46.7)	4 (26.7)	4 (26.7)	0		5 (33.3)	5 (33.3)	1 (6.7)	4 (26.7)	
Diagnosis method												
Only clinical	0	0	0	0	0	0	0.588	0	0	0	0	0.753
Clinical procedures	2 (0.9)	0	0	1 (50.0)	1 (50.0)	0		1 (50.0)	1 (50.0)	0	0	
Cytological	4 (1.8)	0	3 (75.0)	1 (25.0)	0	0		1 (25.0)	2 (50.0)	0	1 (25.0)	
Histological	215 (97.3)	6 (2.8)	119 (55.3)	61 (28.4)	26 (12.1)	3 (1.4)		116 (54.0)	63 (29.3)	13 (6.0)	23 (10.7)	
Clinical stage												
Stage I	16 (7.2)	0	9 (56.2)	5 (31.3)	1 (6.25)	1 (6.25)	0.097	7 (43.8)	6 (37.5)	0	3 (18.8)	0.445
Stage II	36 (16.3)	4 (11.1)	25 (69.5)	7 (19.4)	0	0		16 (44.4)	16 (44.4)	2 (5.6)	2 (5.6)	
Stage III	30 (13.9)	2 (6.7)	13 (43.3)	11 (36.7)	2 (6.7)	2 (6.7)		18 (60.0)	7 (23.3)	3 (10.0)	2 (6.7)	
Stage IV	13 (5.9)	0	7 (53.8)	3 (23.1)	3 (23.1)	0		8 (61.5)	5 (38.5)	0	0	
Unknown	126 (57.0)	0	68 (54.0)	37 (29.3)	21 (16.7)	0		69 (54.8)	32 (25.4)	8 (6.3)	17 (13.5)	
**Lung**	201	15 (7.5)	127 (63.5)	42 (21.0)	18 (8)	1 (0.5)		90 (44.8)	82 (40.8)	17 (8.5)	12 (6.0)	
Sex												
Women	84 ( 41.8)	7 (8.3)	55 (65.5)	14 (16.7)	8 (9.5)	0	0.585	38 (45.2)	32 (38.1)	10 (11.9)	4 (4.8)	0.323
Men	117 (58.2)	8 (6.9)	72 (62.1)	28 (24.1)	8 (6.8)	1 (0.8)		52 (44.4)	50 (42.7)	7 (6.0)	8 (6.8)	
Age, median (95% CI)	66.0 (63.0-67.0)	65.0 (61.2-73.1)	67.0 (63.0-68.0)	64.0 (59.1-68.8)	63.0 (58.1-69.0)	73		64.0 (62.0-67.0)	67.0 (64.0-70.0)	68.0 (63.0-74.0)	62.5 (41.5-68.0)	
0 a 59	59 (29.4)	2 (3.4)	38 (64.4)	15 (25.4)	4 (6.8)	0	0.418	27 (45.8)	24 (40.7)	3 (5.1)	5 (8.5)	0.576
60+	142 (70.6)	13 (9.2)	89 (62.7)	27 (19.0)	12 (8.5)	1 (0.7)		63 (44.4)	58 (40.8)	14 (9.9)	7 (4.9)	
Histology												
Squamous cell Ca.	72 (35.8)	2 (2.8)	52 (72.2)	10 (13.9)	8 (11.1)	0	0.210	32 (44.4)	32 (44.4)	4 (5.6)	4 (5.6)	0.712
Adenocarcinoma	54 (26.9)	4 (7.4)	31 (57.4)	15 (27.8)	3 (5.6)	1 (1.9)		21 (38.9)	21 (38.9)	7 (13.0)	5 (9.3)	
Small cell Ca.	16 (8.0)	1 (6.3)	10 (62.5)	3 (18.8)	2 (12.5)	0		7 (43.8)	6 (37.5)	2 (12.5)	1 (6.3)	
Other and NOS	59 (29.4)	8 (13.6)	34 (57.6)	14 (23.7)	3 (5.1)	0		30 (50.8)	23 (39.0)	4 (6.8)	2 (3.4)	
Diagnostic basis												
Only clinical	3 (1.5)	0	2 (66.6)	1 (33.3)	0	0	0.418	2 (66.7)	1 (33.3)	0	0	0.646
Clinical procedures	16 (8.0)	2 (12.5)	7 (43.75)	7 (43.75)	0	0		9 (56.3)	6 (37.5)	0	1 (6.3)	
Cytological	29 (14.4)	2 (6.9)	16 (55.2)	7 (24.1)	3 (10.3)	1 (3.4)		12 (41.4)	13 (44.8)	1 (3.4)	3 (10.3)	
Histological	153 (76.1)	11 (7.2)	102 (66.6)	27 (17.6)	13 (8.5)	0		67 (43.8)	62 (40.5)	16 (10.5)	8 (5.2)	
**Prostate**	275	21 (7.6)	209 (76.0)	19 (6.9)	22 (8.0)	4 (1.4)		62 (22.5)	136 (49.5)	41 (14.9)	36 (13.1)	
Age, median (95% CI)	71.0 (69.0-72.0)	67.0 (61.4-70.0)	71.0 (70.0.73.0)	73.0 (67.4-74.6)	70.5 (64.9-78.1)	73.5 (65.0-85.0)		71.5 (69.0-74.0)	72.5 (71.0-74.0)	67.0 (65.0-70.0)	68.0 (63.0-72.0)	
0 a 59	43 (15.6)	5 (11.6)	34 (79.1)	3 (7.0)	1 (2.3)	0		7 (16.3)	16 (37.2)	10 (23.3)	10 (23.3)	0.090
60+	232 (84.4)	16 (6.9)	175 (75.4)	16 (6.9)	21 (9.1)	4 (1.7)		55 (23.7)	120 (51.7)	31 (13.4)	26 (11.2)	
Histology												
Adenocarcinoma	257 (93.5)	21 (8.2)	194 (75.5)	18 (7.0)	20 (7.8)	4 (1.6)	0.602	60 (23.3)	128 (49.8)	35 (13.6)	34 (13.2)	0.065
Other and NOS	18 (6.5)	0	15 (83.3)	1 (5.6)	2 (11.1)	0		2 (11.1)	8 (44.4)	6 (33.3)	2 (11.1)	
Diagnosis method												
Only clinical	1 (0.4)	0	0	1 (100.0)	0	0	**0.031**	0	1 (100.0)	0	0	0.633
Clinical procedures	2 (0.7)	0	2 (100.0)	0	0	0		0	1 (50.0)	1 (50.0)	0	
Cytological	0	0	0	0	0	0		0	0	0	0	
Histological	272 (98.9)	21 (7.7)	207 (76.1)	18 (6.6)	22 (8.1)	4 (1.5)		62 (22.8)	134 (49.3)	40 (14.7)	36 (13.2)	
**Stomach**	328	14 (4.1)	184 (56.3)	68 (20.7)	60 (18.3)	2 (0.6)		135 (41.2)	128 (39.0)	29 (8.8)	36 (11.0)	
Sex												
Women	117 (35.7)	4 (3.4)	62 (53.0)	27 (23.1)	23 (19.7)	1 (0.8)	0.732	53 (45.3)	43 (36.8)	13 (11.1)	8 (6.8)	0.433
Men	211 (64.3)	10 (4.7)	122 (57.8)	41 (19.4)	37 (17.5)	1 (0.5)		82 (38.9)	85 (40.3)	16 (7.6)	28 (13.3)	
Age, median (95% CI)	66.0 (63.0-68.0)	63.5 (57.5-76.0)	66.0 (63.0-68.0)	65.0 (58.4-68.0)	67.0 (61.8-72.0)	46.5 (43.0-50.0)		64.0 (61.0-67.0)	65.0 (62.0-69.0)	66.0 (56.0-70.3)	72.0 (67.0-75.4)	
0 a 59	114 (43.8)	5 (4.4)	59 (51.8)	28 (24.6)	20 (17.5)	2 (1.8)		50 (43.9)	43 (37.7)	11 (9.6)	10 (8.8)	0.813
60+	214 (65.2)	9 (4.2)	125 (58.4)	40 (18.7)	40 (18.7)	0		85 (39.7)	85 (39.7)	18 (8.4)	26 (12.1)	
Histology												
AdCa., intestinal	147 (44.8)	8 (5.4)	78 (53.1)	35 (23.8)	25 (17.0)	1 (0.7)		58 (39.5)	60 (40.8)	15 (10.2)	14 (9.5)	0.762
Diffuse Ca.	75 (22.9)	2 (2.79	45 (60.0)	11 (14.7)	16 (21.3)	1 (1.3)		35 (46.7)	24 (32.0)	7 (9.3)	9 (12.0)	
AdCa, others	49 (14.9)	3 (6.1)	33 (67.3)	7 (14.3)	6 (1.2)	0		18 (36.7)	18 (36.7)	4 (8.2)	9 (18.4)	
Other and NOS	57 (17.4)	1 (1.8)	28 (49.1)	15 (26.3)	13 (22.8)	0		24 (42.1)	26 (45.6)	3 (5.3)	4 (7.0)	
(Stomach…)												
Diagnosis method												
Only clinical	2 (0.6)	0	0	2 (100.0)	0	0	**0.042**	2 (100.0)	0	0	0	0.400
Clinical procedures	14 (4.3)	0	5 (35.7)	6 (42.9)	3 (21.4)	0		7 (50.0)	5 (35.7)	0	2 (14.3)	
Cytological	0	0	0	0	0	0		0	0	0	0	
Histological	312 (95.1)	14 (4.5)	179 (57.4)	60 (19.2)	57 (18.3)	2 (0.6)		126 (40.4)	123 (39.4)	29 (9.3)	34 (10.9)	
