# Supplementary material for: Health inequities and cancer survival in Manizales, Colombia: a population-based study
Source: Colomb Med (Cali). 2018 Mar 30;49(1):63–72. doi: 10.25100/cm.v49i1.3629 (PMC6018827; doi:10.25100/cm.v49i1.3629)
Supplement: Related texts

# Research article: Inequidades en salud y supervivencia al cáncer en Manizales, Colombia: un estudio de base poblacional

## Introducción

Las diferencias socioeconómicas en la supervivencia por cáncer han sido bien documentadas en las últimas dos décadas ^1^. Las disparidades en el estadío al momento del diagnóstico han sido consideradas como el factor que causa estas diferencias. Sin embargo, hay evidencia de que el acceso diferencial al tratamiento adecuado y oportuno también determina inequidades en la supervivencia por cáncer según grupos socioeconómicos^1^. Las estimaciones del riesgo relativo de muerte que comparan los grupos más deprimidos con los grupos de mayor bienestar han estado alrededor de 1.3 a 1.5 veces; los factores relacionados a estas diferencias incluyen condiciones del paciente y del tumor, y el acceso y la calidad de la atención^1^. En países con aseguramiento en salud universal como Canadá, la posición socioeconómica (PSE) permaneció asociada con la supervivencia por cáncer, y las diferencias no se debieron al estadío del diagnóstico ^2^. Las desigualdades y las inequidades han sido reportadas para combinaciones de diferentes localizaciones de cáncer ^1^, pero también para localizaciones específicas incluyendo los cánceres de mama ^3^, cuello uterino ^4^, pulmón ^5^, próstata ^6^ y estómago ^7^^,^^8^. Para los cánceres de mama y cuello uterino, se han observado grandes diferencias entre países desarrollados y en desarrollo, pero también entre países desarrollados y al interior de los países ^9^. Para los cánceres de la mujer, el lugar de residencia, el ingreso, el nivel educativo y socioeconómico, la etnia, y el estatus migratorio han estado asociados con inequidades en la supervivencia ^9^.

En Colombia, el aseguramiento en salud financiado a través de contribuciones de los trabajadores y de los empleadores (régimen contributivo) es obligatorio para los empleados dependientes y parcialmente voluntario para los trabajadores independientes. Una pequeña proporción (<5%) de la población que trabaja en ciertos sectores públicos tienen planes especiales o excepcionales de aseguramiento en salud (régimen especial o de excepción). La población pobre esta cubierta por un sistema de aseguramiento subsidiado y financiado a través de los impuestos (régimen subsidiado) ^10^. Para el año 2005, los regímenes contributivo y subsidiado cubrían el 36.3% y el 43.3% de la población, respectivamente, mientras que alrededor del 20% de la población no tenía aseguramiento en salud ^11^. En teoría, los regímenes especial/excepcional y contributivo ofrecen el mejor acceso al cuidado, pero en la práctica los regímenes especial y excepcional han mostrado algunos problemas que podrían hacer que las condiciones de sus afiliados fueran peores que las de aquéllos en el régimen contributivo ^12^^,^^13^. Legislaciones recientes (años 2011^14^ y 2015 ^15^) han abogado por el acceso universal a la atención en salud sin diferencias por la PSE. Sin embargo, estos cambios legislativos no han sido implementados completamente, y los pacientes diagnosticados antes del año 2010 pudieron experimentar tasas de supervivencia diferentes dependiendo de su régimen de aseguramiento en salud (RAS) y de su PSE. Recientemente fueron documentadas grandes disparidades en supervivencia de pacientes con cáncer gástrico en una ciudad en Colombia, a pesar de los avances en la cobertura de aseguramiento ^8^ y, por lo tanto, no sorprende que existan grandes inequidades en la mortalidad por cáncer de base poblacional ^16^^,^^17^. Muy poca información de supervivencia de base poblacional por indicadores socioeconómicos está disponible para otras ciudades y otros cánceres en Colombia.

Manizales es una ciudad andina intermedia en Colombia con una población proyectada en 2005 (año de mitad de período) de 379,794 habitantes ^18^. Desde 2003, la ciudad cuenta con un registro de cáncer de base poblacional que cumple con los estándares de calidad internacionales propuestos por la Agencia Internacional para la Investigación en Cáncer ^19^ (IARC, por su sigla en inglés) y ha reportado tasas de incidencia que son ligeramente superiores que las estimaciones nacionales, principalmente para cánceres de mama, pulmón y estómago ^20^^,^^21^ coincidiendo con una tasa de tabaquismo más alta que el promedio del país y con la ubicación geográfica sobre los Andes. Al conocer la incidencia, hay la necesidad de evaluar la supervivencia de base poblacional para esta población, y evaluar el efecto del RAS y de la PSE sobre las tasas de supervivencia. El objetivo de este artículo fue analizar las diferencias la supervivencia en pacientes con cánceres de mama, cuello uterino, pulmón, próstata y estómago como resultado del aseguramiento en salud y el nivel socioeconómico en una ciudad intermedia en Colombia.

## Materiales and Métodos

### Tipo of estudio

Estudio de cohorte exploratorio de base poblacional.

### Pacientes y seguimiento

Se incluyeron un total de 1,482 pacientes residentes en Manizales con cánceres de mama, cuello uterino, pulmón, próstata y estómago diagnosticados entre 2003 y 2007 y caracterizados en su línea de base por el Registro Poblacional de Cáncer de Manizales -RPC-Manizales- (la información sobre calidad de los datos del RPC-Manizales fue publicada previamente ^19^^,^^22^, así: 380 casos de cáncer de mama en mujeres (se excluyeron 5 casos en hombres), 226 de cuello uterino, 230 de pulmón (ambos sexos), 296 de próstata, y 347 de estómago. Los porcentajes de casos con verificación microscópica fueron 95.8%, 96.9%, 78.6%, 92.5% y 89.4% para mama, cuello uterino, pulmón, próstata y estómago, respectivamente.

Todos los casos fueron seguidos hasta un máximo de 60 meses o hasta diciembre 31 de 2013 a partir de la fecha de diagnóstico para identificar el evento de estudio (muerte debida a todas las causas) y el tiempo al evento a través de apareamiento de números de identificación personal y nombres de los casos incidentes con los registros de estadísticas vitales provistos por la autoridad sanitaria local; además, realizamos una búsqueda manual en los listados del censo electoral y en las bases de datos de aseguramiento en salud. El seguimiento activo fue realizado mediante la consulta de los expedientes clínicos si estaban disponibles. Cuando solo los datos del año estuvieron disponibles, el mes y el día fueron asignados al 30 de junio. Los pacientes sin el evento (muerte) fueron censurados a los cinco años de seguimiento. Los sujetos fueron considerados vivos si se encontraban en los listados electorales o si fueron reportados como “activos” en las bases de datos de aseguramiento hasta diciembre 31 de 2013. El tiempo de supervivencia fue calculado como la diferencia entre la fecha de incidencia y la fecha de muerte, del último contacto con los servicios de salud, de pérdida del seguimiento, o la fecha en que los casos fueron censurados. Para la fecha de incidencia, el RPC-Manizales usa las reglas de la Red Europea de Registros de Cáncer ^23^.

Dieciséis sujetos con diagnóstico solamente clínico quedaron perdidos del seguimiento el día del diagnóstico y, por lo tanto, fueron tratados igual que los casos diagnosticados solo por certificado de defunción (DCO, por su sigla en inglés). Hubo 77 casos DCO, que representaron el 5.2% del total de casos (mama: n= 5 (1.3%); cuello uterino: n= 5 (2.2%); pulmón: n= 29 (12.6%); próstata: n=19 (6.4%) y estómago: n=19 (5.5%)). De acuerdo con los estándares internacionales de registro, un caso es marcado como DCO cuando el certificado de defunción es la única fuente de datos del caso, es decir, no es posible recuperar datos de otras fuentes como reportes de anatomía patológica, imágenes médicas, o registros clínicos. Como los casos DCO por definición no tienen información sobre el tiempo hasta el evento estos fueron excluidos del análisis de supervivencia.

### Características clínicas y demográficas

La información sobre subtipo histológico, codificado de acuerdo con la Clasificación Internacional de Enfermedades para Oncología, 3ª revisión -CIEO-3-, estuvo disponible para el 95.2% de los casos; el estadío clínico al momento del diagnóstico según el sistema TNM estuvo disponible en proporciones útiles para el análisis solamente para los pacientes con cánceres de mama (62%) y cuello uterino (42%). Para las otras localizaciones, el estadío clínico estuvo disponible para menos del 30% de los casos y, por lo tanto, no pudo ser utilizado. No hubo casos con dato perdido para la edad al momento del diagnóstico. Más del 85% de los pacientes tuvieron datos completos para la fecha de nacimiento, y sus edades en los registros clínicos fueron consistente con las edades calculadas; para los pacientes sin fecha de nacimiento, la edad en los registros clínicos fue asumida como correcta.

Se observaron diferencias en el método de diagnóstico solamente para los cánceres de próstata y estómago, en los cuales los diagnósticos “sólo clínico” y “por otros procedimientos clínicos” se presentaron en los pacientes de los regímenes contributivo y subsidiado y en los no asegurados, mientras que el 100% de los pacientes afiliados al régimen especial/excepcional fueron confirmados histológicamente.

### Indicadores socioeconómicos

Las variables para PSE y RAS fueron definidas siguiendo las categorías utilizadas previamente en otra población colombiana ^8^. El estrato socioeconómico (ES) del lugar de residencia al momento del diagnóstico fue usado como indicador de PSE. En Colombia, el ES es definido de acuerdo con las características internas y externas de la vivienda y del barrio, oscilando entre lo puramente funcional e indispensable hasta características estéticas, ornamentales o suntuosas. El ES reportado en categorías del 1 al 6, donde 1 y 2 corresponden al estrato social bajo, 3 y 4 al medio, y 5 y 6 al alto. El RAS a la fecha del diagnóstico fue usado agrupando los regímenes especial y de excepción un una categoría única, además del régimen contributivo, el régimen subsidiado, y un grupo para las personas no afiliadas.

### Análisis estadístico

Se obtuvieron proporciones de supervivencia en diferentes tiempos usando el método de Kaplan-Meier, estratificando por RAS y ES, edad, sexo, subtipo histológico y por estadío clínico al diagnóstico, esta última variable solo para los cánceres de mama y cuello uterino. El supuesto de proporcionalidad para los modelos de Cox fue verificado mediante exploración visual de los gráficos log-log en STATA 12; los supuestos no fueron violados. Se ajustaron tres modelos de regresión multivariada de Cox para cada cáncer y para cada variable de interés, RAS y ES: i) un modelo univariado (nulo); ii) un modelo multivarado “A” con edad, sexo (pulmón y estómago), subtipo histológico, y estadío clínico (mama y cuello uterino) como covariables ; y iii) un modelo multivariado “B” conteniendo todas las variables del modelo A más un término adicional para ES en el modelo de RAS y viceversa ^8^. Todos los cálculos fueron realizados usando STATA®SE versión 12.0.

### Consideraciones éticas

Esta investigación fue aprobada por los Comités de Ética de la Investigación de la Universidad del Valle, de la Universidad de Caldas y del Instituto Nacional de Cancerología de Colombia.

### Resultados

Las características de los pacientes y de los tumores para los 1,405 casos incidentes se muestran en laTabla suplementaria 1S.

Para los cinco tumores estudiados, 1,384 casos fueron finalmente analizados. La pérdida de seguimiento fue de 1.7% para los cinco cánceres combinados (0.8% para mama, 2.3% para cuello uterino, 1.9% para pulmón, 1.8% para próstata, y 2.1% para estómago). En Manizales, la cobertura de aseguramiento en salud entre los pacientes con cáncer fue del 88.8%, con excepción de los pacientes con cáncer gástrico, en quienes el porcentaje de no afiliados alcanzó el 18.3%. Al momento del diagnóstico, los pacientes de cáncer de mama y próstata sin aseguramiento en salud tendieron a ser más viejos que los afiliados a algún régimen, pero las diferencias no fueron estadísticamente significativas.

A los 5 años de seguimiento, se observaron 700 muertes (todas las causas). El tiempo medio de seguimiento fue de 38.4 meses (IC 95%: 37.2-39.7), oscilando desde 18.5 meses para pulmón hasta 50.8 meses para cáncer de mama. La supervivencia observada (SO) fue de 49.1% (IC 95%: 46.4-51.7) para los cinco tumores combinados. Por tipo de cáncer, las proporciones de SO a 5 años fueron 71.0% (IC 95%: 66.1-75.3), 51.4% (44.6-57.9), 15.4% (10.7-20.8), 71.1% (65.3-76.1) y 23.8% (19.3-28.6) para los cánceres de mama, cuello uterino, pulmón, próstata y estómago, respectivamente (Tabla 1).

**Tabla 1:** Estimaciones de supervivencia observada por sitio de cáncer y factores pronósticos. Manizales, 2003-2013.

		Casos (N)	Muertes (n)	Proporción de sobrevida (%)			
				12 m	36 m	60 m	Prueba WBG*
Mama	Todos los casos	375	108	93.8	81.0	71.0	
	Edad al diagnóstico						
	0 a 49	116	29	96.5	85.2	74.8	X^2^=1.63
	50+	258	79	92.6	79.1	69.3	p=0.20
	Histología						
	Cancer adeno	307	82	95.1	83.1	73.3	**X** ^**2**^ **=6.19**
	Otros y NOS	67	26	87.9	71.2	60.4	**p=0.013**
	Estadio clínico						
	Estadio I	25	0	100.0	100.0	100.0	**X** ^**2**^ **=80.76**
	Estadio II	97	5	100.0	96.9	94.9	**p=0.000**
	Estadio III	82	19	100.0	89.0	76.8	
	Estadio IV	29	11	93.1	69.0	55.2	
	Desconocido	141	71	85.0	64.3	49.2	
	Todos los casos	220	105	80.7	62.1	51.4	
Cuello uterino	Edad al diagnóstico						
	0 a 49	101	42	83.0	68.0	58.0	X^2^=2.40
	50+	119	63	78.7	57.1	45.8	p=0.121
	Histología						
	Ca. escamocel.	167	80	80.1	60.7	51.6	X^2^= 1.99
	AdenoCa.	38	16	83.9	70.3	56.8	p=0.369
	Otros y NOS	15	9	79.4	57.8	36.1	
	Estadio clínico						** **
	Estadio I	16	2	100.0	93.8	87.5	**X** ^**2**^ **= 13.75**
	Estadio II	36	16	91.7	69.4	55.6	**p=0.008**
	Estadio III	30	19	80.0	53.3	36.7	
	Estadio IV	13	10	69.2	38.5	23.1	
	Desconocido	125	58	76.3	60.5	52.2	
Pulmon	Todos los casos	198	165	43.6	21.0	15.4	
	Sexo						
	Mujeres	81	67	50.6	28.4	17.3	X2= 3.25
	Hombres	117	98	38.7	15.8	14.1	p=0.071
	Edad al diagnóstico						
	0 a 59	59	46	40.7	27.1	22.0	X2=0.94
	60+	139	119	44.9	18.4	12.5	p=0.331
	Histología						
	Ca. escamocel.	71	54	49.3	22.5	16.9	X2=5.96
	AdenoCa.	53	44	37.3	17.7	13.8	p=0.113
	Ca. cel. pequeñas	16	13	56.3	31.2	18.7	
	Otros y NOS	58	49	38.6	19.3	14.0	
Prostata	Todos los casos	270	78	92.2	78.9	71.1	
	Edad al diagnóstico						
	0 a 59	43	8	95.4	83.7	81.4	*X*2=2.05
	60+	227	70	91.6	77.8	69.2	p=0.152
	Histología						
	AdenoCa.	257	70	92.3	79.4	72.2	*X*2=2.30
	Otros and NOS	18	8	83.3	72.2	55.6	p=0.129
Estómago	Todos los casos	322	244	49.4	33.1	23.8	
	Sexo						
	Mujeres	114	83	57.6	39.0	26.6	X2=1.31
	Hombres	208	161	44.9	30.0	22.2	p=0.252
	Edad al diagnóstico						
	0 a 59	112	86	48.2	32.1	23.2	X2=0.03
	60+	210	158	50.0	33.7	24.1	p=0.857
	Histología						
	Adeno carcinoma intestinal	146	111	54.5	33.8	23.5	X2=2.01
	Carcinoma difuso	73	59	41.7	26.4	18.1	p=0.569
	Adeno Carcinoma, otros	48	35	47.9	37.5	27.1	
	Otros y NOS	55	39	47.3	36.4	29.1	

NOS: Sin otra especificación *WBG: prueba de Wilcoxon-Breslow-Gehan.

Se observaron diferencias estadísticamente significativas en la supervivencia según RAS para los pacientes con cánceres de mama, pulmón, próstata y estómago, con una supervivencia más baja en los pacientes del régimen subsidiado y en los no asegurados. Con relación al estrato socioecocómico, se observaron diferencias significativas solamente para los pacientes con cáncer de pulmón y de próstata, con supervivencia más baja en los pacientes de estratos bajo y medio comparado con el estrato alto, y pacientes de estrato bajo versus pacientes de estratos medio y alto, respectivamente. (Figs. 1 y 2) ^2^. Las proporciones de SO a uno y cinco años fueron significativamente menores en los grupos de pacientes no asegurados o del régimen subsidiado en comparación con los grupos de los regímenes contributivo y especial, con excepción de las pacientes con cáncer de cuello uterino. No obstante, las proporciones de supervivencia para las mujeres con cáncer de cuello uterino y para los pacientes con cáncer de pulmón afiliados al régimen especial/excepcional fueron más bajas que en las demás categorías, incluso más bajas que en la población no afiliada.

**Figura 1:** Supervivencia observada según régimen de aseguramiento en salud y localizaciones de cáncer. Manizales, 2003-2013. WBG: prueba de Wilcoxon-Breslow-Gehan.

**Figura 2:** Supervivencia observada según estrato socioeconómico y localizaciones de cáncer. Manizales, 2003-2013. WBG: prueba de Wilcoxon-Breslow-Gehan.

Como se esperaba, la supervivencia global fue más alta en los pacientes más jóvenes para los cinco sitios estudiados, pero dicha diferencia no fue significativa. De acuerdo con la literatura, el estadío clínico al momento del diagnóstico para los tumores de mama y cuello uterino mostró una fuerte asociación con la supervivencia (Tabla 1 y Fig. 3). La supervivencia fue mejor en las mujeres diagnosticadas con carcinoma ductal de la mama en comparación con otros subtipos histológicos. No se observaron diferencias estadísticamente significativas por subtipo histológico para los demás cánceres. Tampoco se encontraron diferencias en la supervivencia por sexo para los cánceres de pulmón y estómago.

**Figura 3:** Proporción de supervivencia según estadio clínico al diagnóstico para cáncer de mama (basado en 233 casos con estadio clínico conocido) y para cáncer de cuello uterino (basado en 95 casos con estadio conocido). Manizales, 2003-2013.

La Tabla 2 muestra los resultados de los modelos de Cox según RAS y SS por localizaciones de cáncer. Para el cáncer de próstata, las razones de riesgo (Hazard ratio -HR) según RAS permanecieron significativas después de ajustar por edad y subtipo histológico, con menor riesgo de morir para el grupo de régimen especial/excepcional en comparación con el grupo de pacientes del régimen subsidiado (HR: 0.17 (IC 95%: 0.04-0.80)). Estos resultados permanecieron como significativos en el análisis multivariado (modelos A y B). Para cáncer de estómago, los pacientes en el régimen contributivo tuvieron mejor supervivencia tanto en el modelo nulo (univariado) como en los modelos A y B (multivariado), con alrededor de 30% menos riego de morir en comparación con pacientes en el régimen subsidiado. Con relación el estrato socioeconómico, los pacientes con cáncer de próstata de estrato medio mostraron cerca de 47% menos riesgo de morir que los pacientes de estrato bajo (HR 0.53, IC 95%: 0.31-0.88), independientemente del RAS. Las diferencias en los otros tipos de cáncer no alcanzaron la significancia estadística, posiblemente debido al bajo número de casos en cada grupo.

**Tabla 2:** Modelos de riesgos proporcionales (Cox) de sobrevida por sitio del cáncer.

		Análisis univariado		Análisis multivariado			
		HR	IC 95%	Modelo A		Modelo B	
				HR	95% CI	HR	95% CI
**Mama**	RAS *						
	Contributivo	0.77	0.47-12.7	0.71	0.43-1.17	0.77	0.44-1.36
	Especial	0.89	0.38-2.09	0.47	0.20-1.13	0.52	0.20-1.34
	No asegurado	1.66	0.84-2.26	1.27	0.64-2.55	1.41	0.65-3.07
	Casos (eventos)	370 (108)		370 (108)		345 (99)	
	Estrato social ^a^						
	Medio	0.88	0.57-1.35	0.75	0.48-1.17	0.93	0.56-1.55
	Alto	0.61	0.33-1.14	0.65	0.35-1.21	0.84	0.42-1.65
	Casos (eventos)	347 (99)		347 (99)		345 (99)	
**Cuello uterino**	RAS *						
	Contributivo	0.78	0.51-1.22	1.76	0.85-3.64	2.12	0.99-4.55
	Especial	2.20	0.85-5.68	**5.02**	**1.69-14.9**	**7.60**	**1.94-29.7**
	No asegurado	0.92	0.46-1.81	0.94	0.25-3.58	0.90	0.23-3.51
	Casos (eventos)	217 (104)		92 (46)		87 (43)	
	Estrato social ^a^						
	Medio	0.78	0.50-1.22	0.91	0.47-1.76	0.67	0.32-1.41
	Alto	1.20	0.55-2.62	0.84	0.19-3-65	0.38	0.08-1.87
	Casos (eventos)	197 (95)		88 (44)		87 (43)	
**Pulmón**	RAS *						
	Contributivo	0.69	0.47-1.02	0.75	0.51-1.12	0.89	0.58-1.36
	Especial	1.15	0.61-2.19	1.22	0.64-2.32	1.28	0.66-2.49
	No asegurado	1.04	0.54-2.01	1.32	0.66-2.62	1.77	0.83-3.77
	Casos (eventos)	197 (165)		197 (165)		186 (157)	
	Estrato social ^a^						
	Medio	1.00	0.72-1.39	0.99	0.71-1.38	1.03	0.74-1.46
	Alto	0.58	0.32-1.05	0.60	0.33-1.09	0.67	0.36-1.24
	Casos (eventos)	186 (157)		186 (157)		186 (157)	
**Próstata**	RAS *						
	Contributivo	0.58	0.28-1.21	0.56	0.27-1.18	0.56	0.26-1.21
	Especial	**0.17**	**0.04-0.80**	**0.20**	**0.04-0.95**	**0.12**	**0.02-0.99**
	No asegurado	0.89	0.33-2.37	0.74	0.27-2.02	0.81	0.27-2.41
	Casos (eventos)	266 (77)		266 (77)		233 (70)	
	Estrato social ^a^						
	Medio	**0.52**	**0.31-0.87**	**0.53**	**0.31-0.88**	**0.54**	**0.32-0-92**
	Alto	0.63	0.32-1.26	0.71	0.36-1.44	0.78	0.38-1.60
	Casos (eventos)	237 (71)		237 (71)		233 (70)	
Estómago	RAS *						
	Contributivo	**0.72**	**0.52-0.98**	**0.71**	**0.52-0.98**	**0.70**	**0.51-0.98**
	Especial	0.70	0.36-1.38	0.72	0.36-1.41	0.75	0.38-1.50
	No asegurado	0.93	0.62-1.39	0.93	0.62-1.39	1.01	0.65-1.56
	Casos (eventos)	320 (243)		320 (243)		289 (221)	
	Estrato social ^a^						
	Medio	0.80	0.61-1.06	0.80	0.60-1.05	0.82	0.62-1.09
	Alto	0.75	0.46-1.20	0.76	0.47-1.23	0.89	0.55-1.45
Casos (eventos)	291 (222)		291 (222)		289 (221)	

* RAS: Régimen de Aseguramiento en Salud; régimen subsidiado como referencia. a Estrato socioeconómico bajo como referencias. Los modelos fueron ajustados por edad, sexo (pulmón y estómago), subtipo histológico, y estadio clínico al diagnóstico (mama y cuello uterino).

Como era de esperarse, los estadios clínicos avanzados para cáncer de mama y de cuello uterino tuvieron menor supervivencia (HR mayores a 1.0). En concordancia con los resultados del análisis por el método de Kaplan-Meier, las pacientes con cáncer de cuello uterino afiliadas al régimen especial tuvieron riesgos mayores que las mujeres afiliadas al régimen subsidiado, después de ajustar por edad, subtipo histológico y estadio clínico.

El modelo B mostró que la inclusión de ambos términos, RAS y ES, en el mismo modelo modificó las estimaciones de riesgo (HR) en los cinco cánceres combinados y por localizaciones específicas de cáncer, indicando que hay efectos independientes del RAS y del ES sobre la supervivencia.

## Discusión

Este estudio de base poblacional en Manizales demostró diferencias significativas en la supervivencia observada. Las diferencias según régimen de aseguramiento en salud variaron desde 8 puntos porcentuales en los pacientes con cáncer de estómago hasta 32 puntos porcentuales en los pacientes con cáncer de próstata, con peores proporciones de supervivencia para los pacientes del régimen subsidiado y no asegurados. Los riesgos de morir (Hazard Ratios) estimados para RAS estuvieron en concordancia con los reportados por otros estudios ^1^. Las diferencias absolutas según estrato socioeconómico fueron menos notorias, con diferencias entre los estratos bajo y alto de cerca de 16 puntos porcentuales para cáncer de próstata, 10 para mama, 14 para cuello uterino, 5 para pulmón y 11 puntos porcentuales para los pacientes con cáncer de estómago. La magnitud de estas disparidades es similar a la encontrada en EE.UU durante el último cuarto del siglo pasado ^24^.

El sistema de salud colombiano fue radicalmente modificado a finales del siglo XX, resultando en un incremento sustancial de la cobertura del aseguramiento en salud; esta alcanzó casi el 100% de la población alrededor del año 2010. En el papel, esto significó un mejoramiento del acceso a los servicios de salud. 

El acceso real y oportuno a la atención en salud para el diagnóstico y tratamiento del cáncer continúa siendo problemática. Esto se debe particularmente por el alto gasto de bolsillo y los largos períodos de espera para obtener las autorizaciones para el uso de los servicios oncológicos. En Colombia, el acceso a la atención en salud es diferencial y depende del régimen de aseguramiento en salud. Persisten inequidades entre los tipos de afiliación. 

Investigadores locales han resaltado que la universalidad en nuestro sistema de salud no ha sido alcanzada y que hay un estancamiento en materia de acceso a los servicios y en la igualdad ^25^. Adicionalmente, se han descrito enormes disparidades regionales dentro del territorio colombiano, un país que tiene una de las peores distribuciones del ingreso per cápita en el mundo ^26^.

En general, la supervivencia observada a cinco años por sitio del cáncer estuvo por debajo de los observado en la mayoría de los países participantes de los estudios CONCORD ^27^ y EUROCARE ^28^. Las disparidades por RAS y ES para los pacientes con cáncer de mama, próstata y estómago fueron similares a las reportadas en la literatura ^29^^-^^33^.

La proporción de supervivencia en mujeres con cáncer de cuello uterino fue tres veces más baja en aquellas afiliadas al régimen especial/excepcional de salud en comparación con los otros regímenes. Estos resultados inesperados -los regímenes especial y de excepción tienen, en teoría, el plan de atención en salud más generoso- están en línea con la observación de un peor estadio clínico al diagnóstico en este grupo, lo cual sugiere que los programas de detección temprana y tamizaje no están funcionando adecuadamente en las entidades que administran estos regímenes. Con respecto al ES, las tasas de supervivencia de las pacientes con cáncer de cuello uterino fueron 7 y 14 puntos porcentuales más altas en los estratos bajo y medio, respectivamente, en comparación con el estrato alto. Las tasas de incidencia fueron menores en el grupo económicamente más favorecido (estrato alto), y la relativa baja frecuencia de este cáncer en la población más rica puede resultar en una menor percepción del riesgo y en menores tasas de participación en los programas de tamizaje y detección/tratamiento oportunos para el cáncer cervical. Sin embargo, las diferencias en el estadio clínico al diagnóstico no alcanzaron la significancia estadística, quizás debido a que el número de casos en el estrato alto fue muy bajo (Tabla 1S). Al respecto, Brookfield *et al*. ^34^, encontraron que, en mujeres residentes del estado de Florida (EE.UU), el estado de aseguramiento en salud, el estadio y el grado del tumor, y el tratamiento fueron los predictores independientes de desenlaces menos favorables; ni la etnia, ni la posición socioeconómica fueron predictores independientes de desenlaces adversos. Similarmente, Niu *et al.* ^35^, tampoco encontraron diferencias significativas en la supervivencia por cáncer cervical según aseguramiento en salud en mujeres de New Jersey.

Para cáncer de pulmón, se observaron tasas de supervivencia 2.3 y 2.0 veces mayores en los pacientes de los regímenes contributivo y subsidiado y en los no asegurados en comparación con los del régimen especial. Estos resultados son contradictorios con los reportados en EE.UU ^36^ donde los pacientes no asegurados o cobijados por*Medicaid*tuvieron peor supervivencia que aquéllos con aseguramiento privado. Este patrón podría estar reflejando barreras para el diagnóstico y tratamiento tempranos en este subgrupo, que en Manizales está compuesto mayoritariamente por profesores y miembros de la Policía y el Ejército. No obstante, esto debe ser confirmado mediante estudios con un mayor número de pacientes.

Las proporciones de supervivencia fueron alrededor de cinco puntos porcentuales más bajas en el grupo más pobre en comparación con el grupo más rico, lo cual es consistente con las cifras reportadas por Ou *et al* ^37^.

Las disparidades en la supervivencia por cáncer relacionadas con el sistema de salud pueden ser atribuidas a barreras y retrasos para obtener la atención para el diagnóstico, lo cual se asocia con estadios más avanzados al momento del diagnóstico. En Colombia, prácticamente todos los procedimientos médicos requieren autorización del asegurador, lo que en muchos casos lleva a retrasos considerables en la confirmación diagnóstica y en el tratamiento y, consecuentemente, a estadios diagnósticos más avanzados y peores resultados finales ^38^^,^^39^. Por esta razón, muchas personas recurren al pago de bolsillo de algunos servicios para evitar retrasos, pero las personas con bajos recursos financieros no tienen otras vías para acceder a diagnóstico y tratamiento oportunos y están sujetos a los procedimientos administrativos de sus aseguradoras.

### Fortalezas y debilidades

La naturaleza poblacional de este trabajo minimiza el sesgo de selección en las estimaciones y sirve como una herramienta para que los encargados de la política evalúen el acceso y la calidad de los servicios de salud. Aunque los números de pacientes son relativamente bajos debido al tamaño poblacional de Manizales, las estimaciones presentadas son suficientemente robustas para discernir patrones generales.

Una limitación importante de este estudio es la falta de estimaciones de supervivencia relativa por RAS y ES debido a la falta de datos disponibles sobre la población y las tablas de vida desagregadas por estas variables. El RPC-Manizales no tuvo acceso a los datos individuales de causa de muerte, siendo imposible calcular la supervivencia por causa de muerte específica. Para los cánceres de un pronóstico pobre esto no es problemático en tanto la mayoría de los pacientes morirán por su cáncer. Sin embargo, para los cánceres de buen pronóstico (por ejemplo, mama y próstata), una proporción importante de las muertes pudieron deberse a otras causas. Proporciones considerables de los casos tuvieron dato perdido del ES, especialmente en los cánceres de cuello uterino, próstata y estómago (12.2%, 13.1% y 11.0%, respectivamente). Además, el porcentaje de casos con dato perdido para el estadio clínico al diagnóstico fue alto para todos los cánceres, con excepción del de mama y cuello uterino: más de 70% de los casos de cáncer de pulmón, próstata y estómago no tenían información sobre el estadio clínico, lo que impidió incluir esta variable en el análisis multivariado.

El número de casos fue muy bajo en algunas localizaciones de cáncer lo que afectó el análisis. Cerca de 5% de los pacientes fueron excluidos porque tenían tiempo de seguimiento nulo (casos DCO y pérdidas de seguimiento en la fecha del diagnóstico), lo cual teóricamente puede influenciar los resultados en tanto se espera que los casos DCO estén concentrados en los grupos socioeconómicos más pobres. Sin embargo, un artículo reciente mostró que aunque el diagnóstico solo por certificado de defunción esta asociado con un bajo nivel socioeconómico, la exclusión o inclusión de los casos DCO no tuvo impacto significativo sobre las estimaciones del riesgo instantáneo (HR) para la supervivencia por variables socioeconómicas ^40^.

## Conclusiones

En Manizales existen importantes inequidaes en la supervivencia por cáncer relacionadas con el aseguramiento en salud y con la posición socioeconómica. Las diferencias pueden ser atribuidas a inequidades en el perfil de comorbilidades, en el estadio clínico al diagnóstico, o en barreras para el acceso oportuno al tratamiento efectivo como lo indican las diferencias observadas entre los regímenes de aseguramiento.
